# Endometrial cancer prognosis correlates with the expression of L1CAM and miR34a biomarkers

**DOI:** 10.1186/s13046-018-0816-1

**Published:** 2018-07-06

**Authors:** Giacomo Corrado, Valentina Laquintana, Rossella Loria, Mariantonia Carosi, Laura de Salvo, Isabella Sperduti, Ashanti Zampa, Lucia Cicchillitti, Giulia Piaggio, Giuseppe Cutillo, Rita Falcioni, Enrico Vizza

**Affiliations:** 1 Department of Experimental Clinical Oncology, Gynecologic Oncology Unit, IRCCS - Regina Elena National Cancer Institute, Via Elio chianesi, 53 –, 00144 Rome, Italy; 2 Department of Research, Advanced Diagnostics and Technological Innovation, Area of Translational Research, IRCCS - Regina Elena National Cancer Institute, Rome, Italy; 3 Department of Research, Advanced Diagnostics and Technological Innovation, Anatomy Pathology Unit, IRCCS - Regina Elena National Cancer Institute, Rome, Italy; 4 Scientific Direction, IRCCS - Regina Elena National Cancer Institute, Rome, Italy; 5 Present address: Department of Women and Children Health, Gynecologic Oncology Unit, Fondazione Policlinico Universitario A. Gemelli – IRCCS, Università Cattolica del Sacro Cuore, Roma, Italy

**Keywords:** L1CAM, Endometrial cancer, Prognostic biomarker, Personalised approach, Innovative biotechnology

## Abstract

**Background:**

Patients with endometrial cancer (EC) and presumably with good prognosis may develop a recurrence indicating that the classification of this tumor is still not definitive and that new markers are needed to identify a subgroup at risk of relapse. The cell adhesion molecule L1CAM is highly expressed in several human carcinomas and has recently been described as a new marker for endometrial and ovarian carcinomas. The aim of this study was to determine the relevance of L1CAM in recurrent EC.

**Methods:**

In this work we have analyzed, by immunohistochemical and RT-qPCR analysis, the expression of L1CAM in a cohort of 113 endometrial cancers at different stages, which 50% have relapsed. As a predictor of good outcome, the tumors were also analyzed for the expression of miR-34a, a post-transcriptional regulator of L1CAM.

**Results:**

Among metastatic EC, the highest levels (60%) and the median level (24%) of L1CAM in tumors correlate with the progression, suggesting that the expression of this molecule is linked to the tumor component most involved in metastatic processes. We also found an inverse correlation between miR-34a and L1CAM protein expression, suggesting that miR-34a is a positive prognostic marker of EC.

**Conclusions:**

Our results demonstrate the expression of L1CAM and miR-34a in EC as prognostic factors that identify subgroup of patients at high risk of recurrence suggesting for them more aggressive schedules of treatment.

**Electronic supplementary material:**

The online version of this article (10.1186/s13046-018-0816-1) contains supplementary material, which is available to authorized users.

## Background

Endometrial cancer (EC) is the most common gynecologic malignancy in industrialized countries [1]. From the pathogenic point of view, EC falls into two different types, so-called types I and II, and this classification subsequently has shown systematic differences in molecular features, and consequently, in prognosis and treatment [2]. Within type I EC, or estrogen-dependent endometrioid, the phosphatidylinositide 3-kinases (PI3K)/protein serine-threonine kinase AKT pathway is the most frequently altered, with inactivation of the phosphatase and tensin homolog tumor (PTEN) suppressor gene, which modulates cell division and enables apoptosis. The most common molecular alterations observed in type 2 EC that consists of the estrogen-independent non-endometrioid carcinoma are p53 and p16 mutations, HER2 overexpression or amplification and loss of E-cadherin [3]. The Cancer Genome Atlas (TCGA) Research Network proposed an innovative classification of EC that divided EC into four categories, with probable influence on treatment recommendations, based on an integrated genomic, transcriptomic and proteomic characterization [4]. Despite these important innovations to better-characterized EC, no effective biomarkers currently exist to direct treatment (adjuvant radiation and/or chemotherapy) in EC, or to triage pelvic and para-aortic lymphadenectomy. In the last years, L1-cell adhesion molecule (L1CAM), a transmembrane protein of the immunoglobulin family that has been implicated in promoting tumor cell proliferation, migration, invasion, and metastasis, has been investigated in EC [5]. The immunohistochemical (IHC) detection of L1CAM in endometrial tumor samples seems to be able to discriminate a subset of highly aggressive tumors with high risk of distant recurrences [6] and to assess the risk of pelvic lymph-node involvement [7]. Moreover, it has been previously reported that miR34-a controls the expression of L1CAM inducing its mRNA degradation [8]. Specifically, it was demonstrated that the overexpression of miR-34a in EC cell line abrogates L1CAM expression and, as consequence, inhibits cell migration [8].

We therefore set out to determine the relevance of L1CAM and miR34-a in recurrent EC, which includes both type <1B and ≥ 1B.

## Methods

### Patients cohort

Retrospective cohorts of formalin-fixed paraffin embedded (FFPE) specimens, derived from 113 patients with EC surgically treated at the Regina Elena National Cancer Institute, were collected from 24/12/2001 to 05/12/2014. The patients of the two cohorts (57 with recurrent and 56 non-recurrent) were selected from a large database with propensity score matching method. The two subsets were matched for potentially confounders: age, stage, grading and histotype. The study was reviewed and approved by the ethical committee of “Regina Elena” National Cancer Institute, and informed consent was obtained from all patients.

### Quantitative RT-PCR

Total RNA, derived from EC, was isolated by PureLink FFPE kit (Invitrogen), and reverse-transcribed using PrimeScript RT reagent kit (Takara). Quantitative PCR (RT-qPCR) was performed using SYBR Green on an ABI Prism 7500 apparatus (Applied Biosystems, Glasgow, UK) in 2 independent experiments in triplicate. The comparative threshold (ΔCt) method was used.

Primer sequences to perform RT-qPCR were:

- L1CAM: Fw-5’ACGAGGGATGGTGTCCACTTCAAA,
- Rev-5’ TTATTGCTGGCAAAGCAGCGGTAG
- GAPDH: Fw-5’ TCCCTGAGCTGAACGGGAAG,
- Rev-5’GGAGGAGTGGGTGTCGCTGT

### Antibody and immunohistochemistry

The mouse monoclonal antibody anti-L1CAM, clone UMAB48, was purchased from OriGene Tchnologies (Rockville, MD, USA). The formalin fixed paraffin-embedded (FFPE) tissue blocks were collected and cut into 5 μm sections and mounted on Superfrost slides. Antigen retrieval was performed at 96 °C (10 mM/L citrate buffer, pH 6) for 20 min. Sections were incubated with the primary antibody anti-L1CAM (1:30) for 30 min at room temperature. Bond Polymer Refine Detection Kit revealed immunoreaction according to manufacturer’s procedure (Leica Biosystems) in an automated autostainer Bond III Leica Biosystems. Diaminobenzidine was used as chromogenic substrate. Microscope Nikon ECLIPSE 55i with digital camera HESP Technology was used. Scale bars 50 μm.

### MicroRNA analysis

Reverse transcription and qRT-PCR amplification were performed in two steps. In the first reverse transcription step, 10 ng of RNA was used in reactions with specific stem-loop RT primer for miR-34a and endogenous control primer for small nuclear RNA U6. Reaction was performed with TaqMan MicroRNA Reverse Transcription Kit, according to the manufacturer’s protocol (Applied Biosystems, Foster City, CA). In the second step, cDNA samples were amplified in Real Time PCR instrument 7500 (Applied Biosystems) with the specific TaqMan miR-34a assay and small nuclear RNA U6 as endogenous control previous tested to verify the minimal variation found among the tissues analyzed. The relative quantity (RQ) of each miRNA was calculated by the comparative CT (2-ΔΔCT) method.

### Statistical analysis

Descriptive statistics were used to describe the patients’ characteristics. Continuous variables are presented as median and range. Proportions are presented as numbers and percentages. Chi square test or Fisher’s exact test, when appropriate, were used to estimate all association between categorical variables. Comparison between groups of continuous variables was performed by the use of Mann-Whitney U test. Receiver operating characteristic (ROC) analysis was performed to find the optimal cut-off for L1CAM expression and mRNA, capable of splitting patients into groups with different recurrence probabilities. Disease Free Survival (DFS) curves were estimated by the Kaplan-Meier product-limit, from the date of surgery until recurrence or last follow-up. The log-rank test was used to assess differences between subgroups. Significance was defined at the *p* ≤ 0.05 level. A multivariate Cox proportional hazard model was developed using stepwise regression (forward selection). Variables testing significant at the univariate analysis were entered into the model, enter limit and remove limit were *P* = 0.10 and *P* = 0.15, respectively. The variables considered at univariate analysis included: age, tumor histology, grading, stage, Body Max Index (BMI), comorbidity (hypertension, diabetes), washing, lymph nodes, lymph vascular space invasion (LVSI), L1CAM expression. SPSS software (SPSS version 21.0, SPSS Inc., Chicago, Illinois, USA) and MedCalc® (10.0.1) statistical programs was used for all analyses.

## Results

A group of 113 EC patients (56 non-recurrent and 57 recurrent EC), median age 67 years (range 40–88) were included in this study and evaluated for DFS. The patients were stratified based on histology, grade of differentiation, FIGO stage, adjuvant therapy, lymph node positivity, washing, LVSI, hypertension, diabetes, and BMI (Table 1). The expression level of L1CAM protein was analyzed, by IHC analysis, in specimens derived from patients surgically treated at the “Regina Elena” National Cancer Institute between 2001 and 2014. Based on the ROC curve, optimal cut-off was determined at 20% for L1CAM (Additional file 1: Figure S1).

**Table 1:** Clinico-pathological characteristics of patients with endometrial cancer

Clinicopathological characteristics	No recurrence	Recurrence
	N (%)	N (%)
Age at diagnosis		
median (range)	65 (40–84)	68 (48–88)
≤ 67 yrs	34 (60.7)	27 (47.4)
> 67 yrs	22 (39.3)	30 (52.6)
BMI		
median (range)	28 (18–53)	31 (21–80)
Grade		
G1	1 (1.8)	6 (10.6)
G2	19 (33.9)	17 (29.8)
G3	36 (64.3)	34 (59.6)
Histology		
Adenocarcinoma (ADK)	46 (82.1)	46 (80.7)
Others	10 (17.9)	11 (19.3)
Stage		
≤ IB	21 (37.5)	32 (56.1)
≥ IB	35 (62.5)	25 (43.9)
Adjuvant treatment		
None	11 (19.6)	15 (26.3)
Radiotherapy	30 (53.6)	22 (38.6)
Chemotherapy	8 (14.3)	11 (19.3)
Radiation and chemotherapy	7 (12.5)	9 (15.8)
Lymph nodes		
Positive	7 (12.5)	6 (10.5)
Negative	49 (87.5)	51(89.5)
Washing		
Positive	7 (12.5)	6 (10.5)
Negative	49 (87.5)	51 (89.5)
LVSI		
Positive	13 (23.2)	13 (22.8)
Negative	43 (76.8)	38 (66.7)
Hypertension		
Yes	27 (48.2)	33 (57.9)
No	29 (51.8)	24 (42.1)
Diabetes		
Yes	6 (10.7)	9 (15.8)
No	50 (89.3)	48 (84.2)

### L1CAM is higher expressed in recurrent compared to non-recurrent EC

**Fig. 1:**
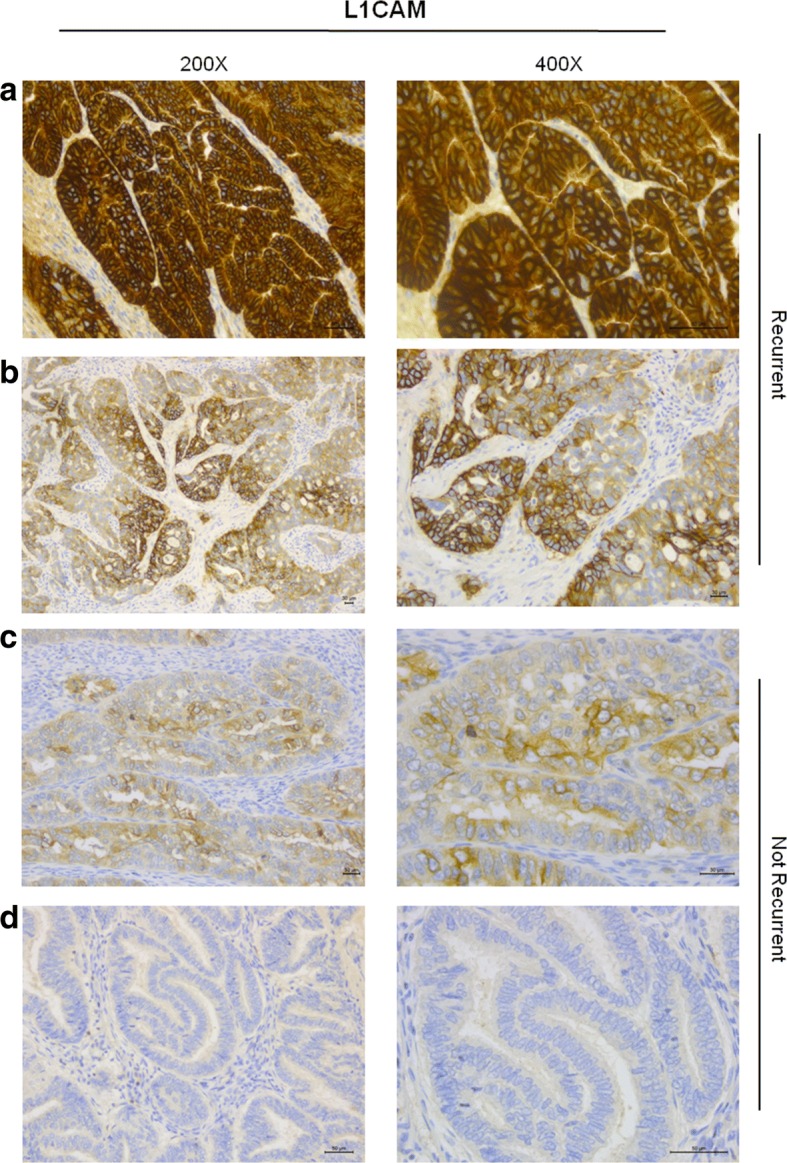
Representative immunohistochemical staining of L1CAM in recurrent and non-recurrent EC. FFPE tumor tissues derived from recurrent EC displaying a strong (score 3+) (**a**) and a moderate (score 2+) (**b**) L1CAM positivity, respectively. FFPE tumor tissues derived from no recurrence EC displaying a mild focal positivity (score 2+ in the 20% of tumor cells) (**c**) and no L1CAM immune-reactivity (**d**). Scale bar 50 μm

Among the recurrent EC samples analyzed, derived from FFPE tissues, we found 14 (24%) of tumors with ≤20% of positive cells, 34 (60%) with >20% of positive cells, and 9 (16%) negative tumors; among the non-recurrent EC analyzed samples we found and 19 (34%) with ≤20% of positive cells, 13 (23%) with >20% of positive cells and 24 (43%) negative tumors (Table 2). Figure 1a-d shows representative IHC analysis of L1CAM on recurrent (a-b) and non-recurrent (c-d) EC patient specimens, scored semi-quantitatively based on staining intensity (0, 1+, 2+, and 3+) and on the % of positivity.

**Table 2:** L1CAM expression in recurrent and no recurrent EC

	n (%)	L1CAM expression n (%)		n (%)	
	Negative	≤ 20% of tumor cells	> 20% of tumor cells		*P* value
Recurrence	9 (16)	14 (24)	34 (60)	57	
No recurrence	24 (43)	19 (34)	13 (23)	56	< 0.0001

### L1CAM expression correlates with lower DFS

The median follow-up of the entire population was 48 months (range 1–162), the median DFS was 48 months (CI95% 14–81). In the univariate analysis we found significant L1CAM expression, age, BMI, and LVSI and number of lymph node.

**Fig. 2:**
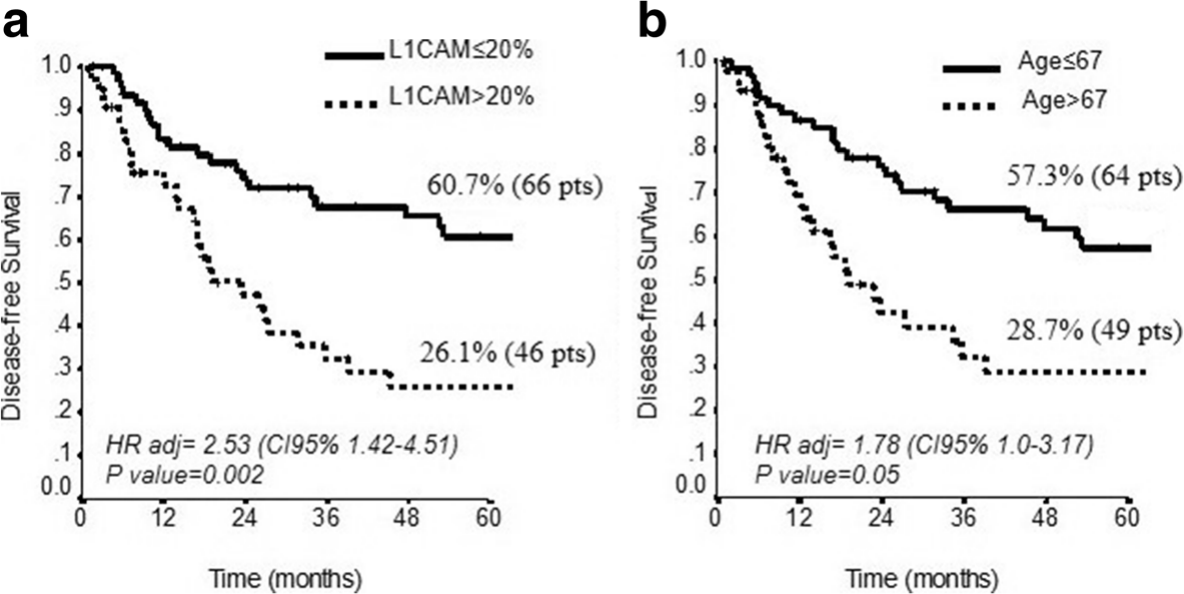
The DFS according to L1CAM expression and age of patients. Kaplan-Meier estimate DFS for (**a**) L1CAM expression (≤20% vs >  20% of positive cells, respectively) (*P* = 0.002), and (**b**) age of the patients (≤67 vs > 67) (*P* = 0.05)

**Fig. 3:**
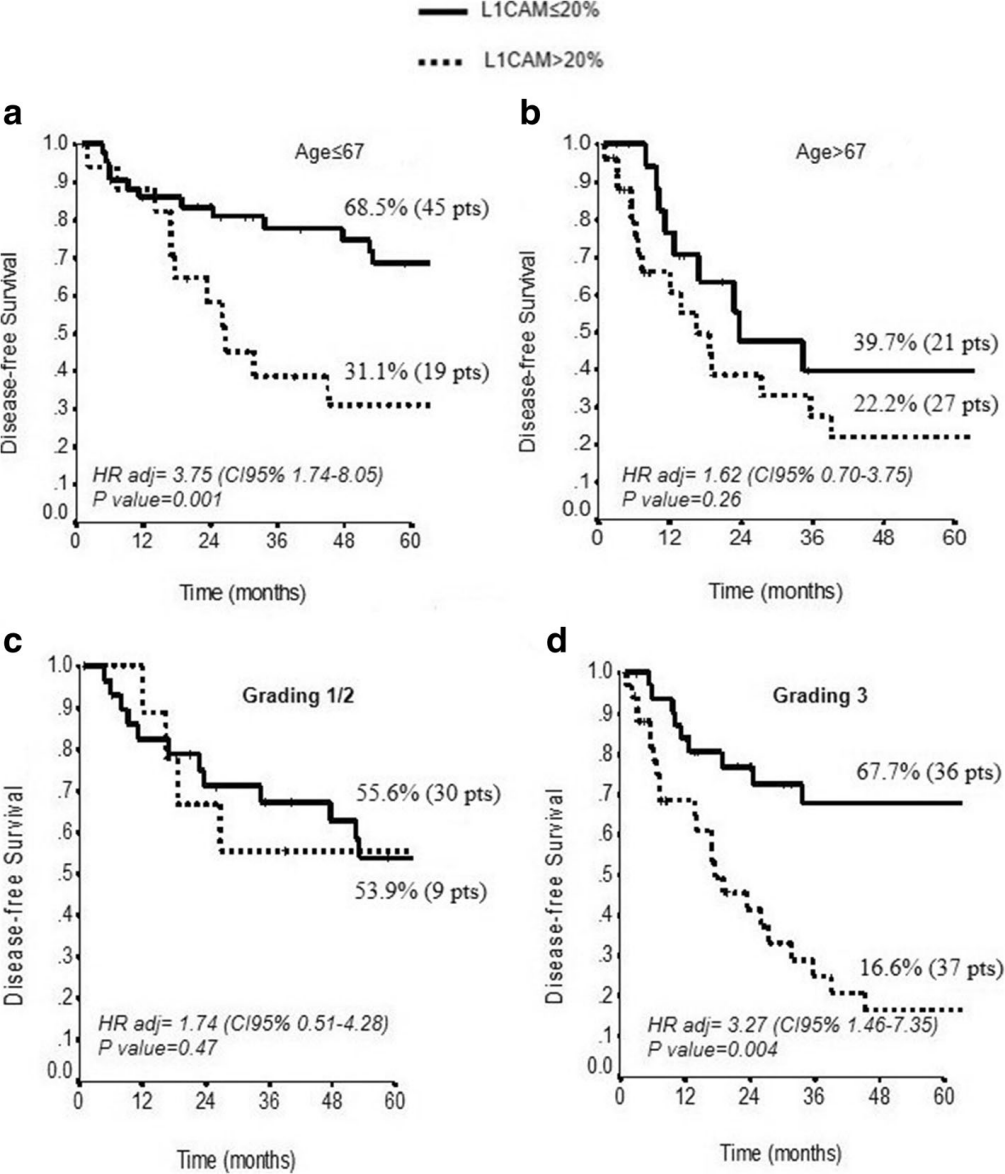
The graph is representing DFS of L1CAM expression in patients of different age and tumor grade. **a**-**b** Kaplan-Meier estimates DFS for L1CAM expression stratified for age of the patients (age ≤ 67 years and age > 67 years). **c**-**d** Kaplan-Meier estimate DFS for high vs low L1CAM expression stratified for grading (grading 1/2 and grading 3). *P*-values were calculated using the log-rank test

At multivariate analysis only L1CAM (HR 2.53, CI95% 1.42–4.51, *p* = 0.002) and age > 67 years (HR 1.78, CI95% 1.0–3.17, *p* = 0.05) were confirmed as significant predictors of worse DFS (Table 3). When we correlated the expression of L1CAM in EC with DFS, we found that higher expression of L1CAM was present in tumor patients with lower disease free survival (5y-DFS 26.1% vs 60.7%, p = 0.002) (Fig. 2a). Furthermore, the correlation between age of patients and DFS highlighted those patients older than 67 years with worse survival (Fig. 2b). Even though we did not observe a significant correlation between L1CAM expression and age of patients (*p* = 0.19), we observed a subgroup of patients with low L1CAM expression and age lower than 67 years at low risk of recurrence compared to a subgroup of patients with low L1CAM expression and age higher than 67 years with an increase of risk of recurrence (5-yrs DFS: 68.5 and 39.7%, respectively) (Fig. 3a, b). When we stratified the expression of high L1CAM with the grading of the disease, we found that higher L1CAM expression predicted a significant shorter DFS in the subgroup of patients with G3 tumor (5y-DFS, 67.7% vs 16.6%, *P* = 0.004) (Fig. 3d); the expression of L1CAM did not predict risk of recurrence in G1/2 patients (*p* = 0.47) (Fig. 3c).

**Table 3:** Univariate and Multivariate analysis for Disease Free Survival

Variables	Disease-free Survival			
	Univariate analysis		Multivariate Analysis	
	HR (CI95%)	*P* value	HR (CI95%)	*P* value
*Age (> 67* vs *≤ 67)*	2.32 (1.32–4.07)	0.003	1.78 (1.0–3.17)	0.05
*Histotype (Other* vs *adenoc.)*	1.41 (0.71–2.83)	0.33	–	NS
*Grading (3* vs *1–2)*	1.45 (0.82–2.58)	0.21	–	NS
*Stage (>IB* vs *≤ IB)*	1.48 (0.81–2.69)	0.20	–	NS
*L1CAM (> 20* vs *≤ 20)*	2.95 (1.69–5.14)	< 0.0001	2.53 (1.42–4.51)	0.002
*BMI*	1.78 (1.03–3.09)	0.04	–	NS
*Comorbidity (yes* vs *no)*	1.29 (0.73–2.27)	0.38	–	NS
*Washing (yes* vs *no)*	1.12 (0.58–2.19)	0.73	–	NS
*LVSI (yes* vs *no)*	2.0 (1.12–3.53)	0.02	–	NS
*Number of lymph nodes*	0.98 (0.95–0.99)	0.04	–	NS

**Fig. 4:**
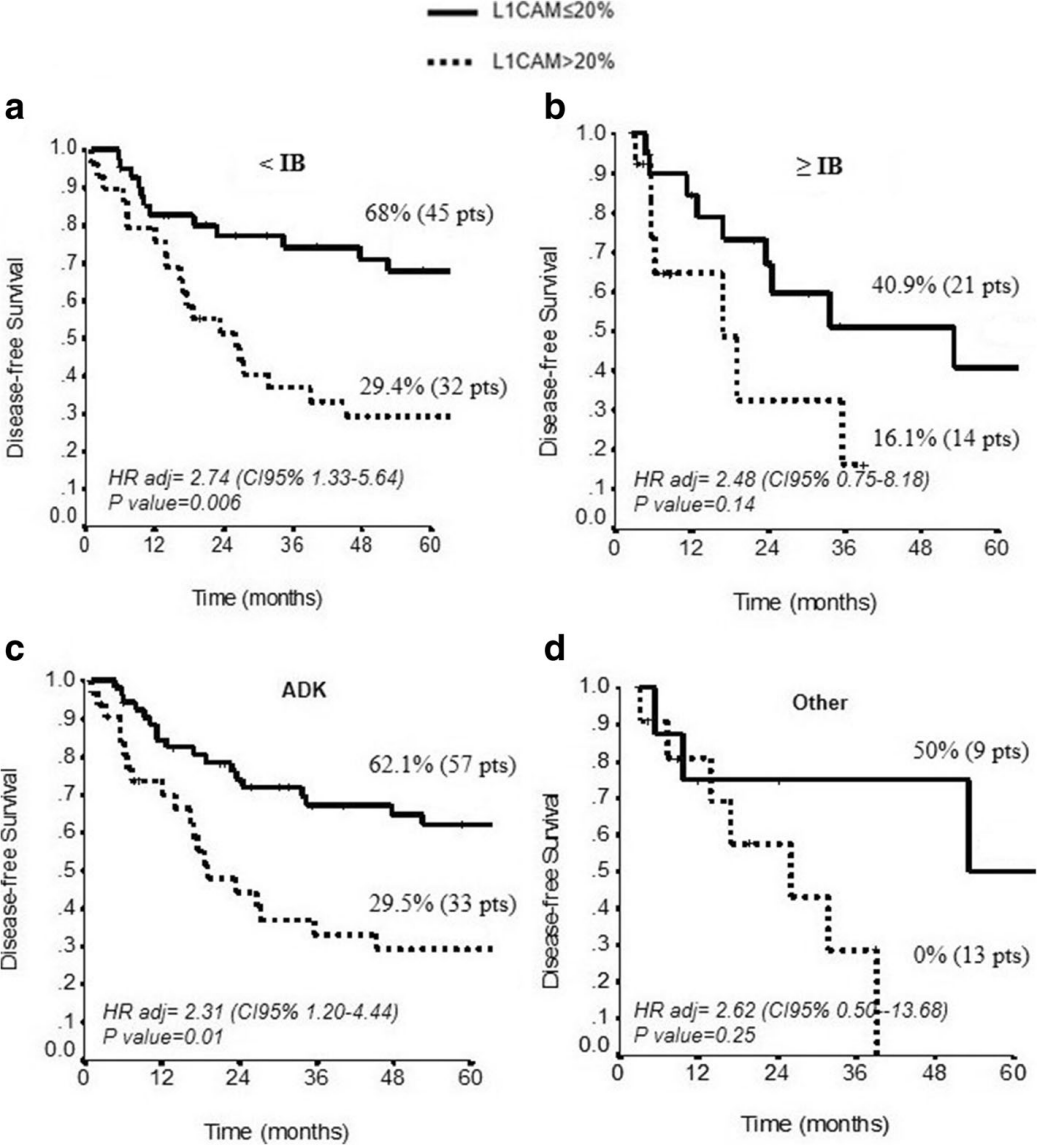
DFS of L1CAM expression in patients of different tumor stage and histology. **a**-**b** Kaplan-Meier estimate DFS for high vs low L1CAM expression stratified in combination with type of tumors (≤IB and > IB) (*P* = 0.006 vs *p* = 0.14); and (**c-d**) histotype (ADK vs other) (*P* = 0.01 vs *p* = 0.25)

When we stratified the L1CAM expression according to the stage of the tumor (<1B vs ≥ 1B) and the histotype (ADK) we found that high level of L1CAM were associated with lower DFS in patients with stage of tumor <1B and G3 grading (*p* = 0.006 and *p* = 0.01, respectively) (Fig. 4a and c); the expression of L1CAM did not predict risk of recurrence in patients with stage of the tumor ≥1B or in not EC (*p* = 0.14 and *p* = 0.25, respectively) (Fig. 4b and d).

### High expression of miR-34a is a marker of good prognosis in EC

**Fig. 5:**
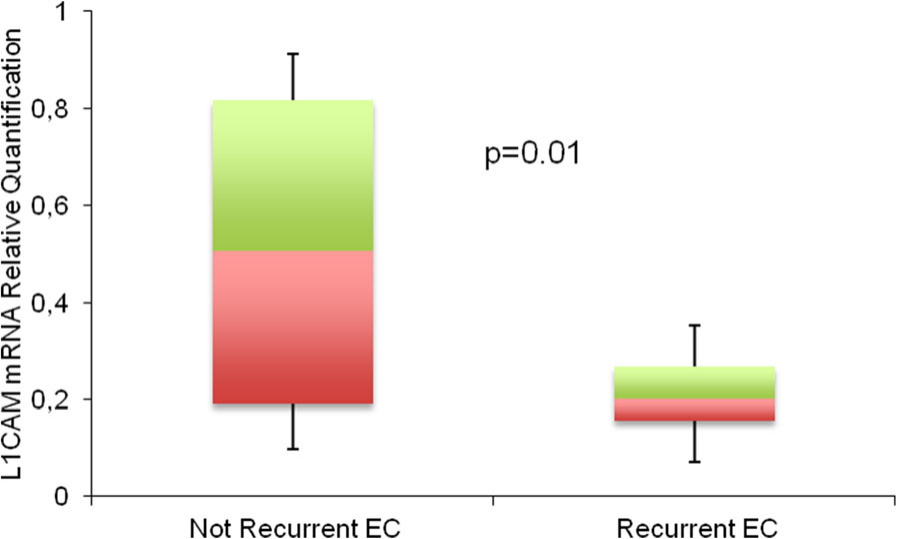
L1CAM expression at the mRNA level on the cohort of 113 EC patients. Expression of L1CAM mRNA was examined by qRT-PCR on specimens derived from patients with no recurrence vs recurrent EC tumors (*P* = 0.01)

**Fig. 6:**
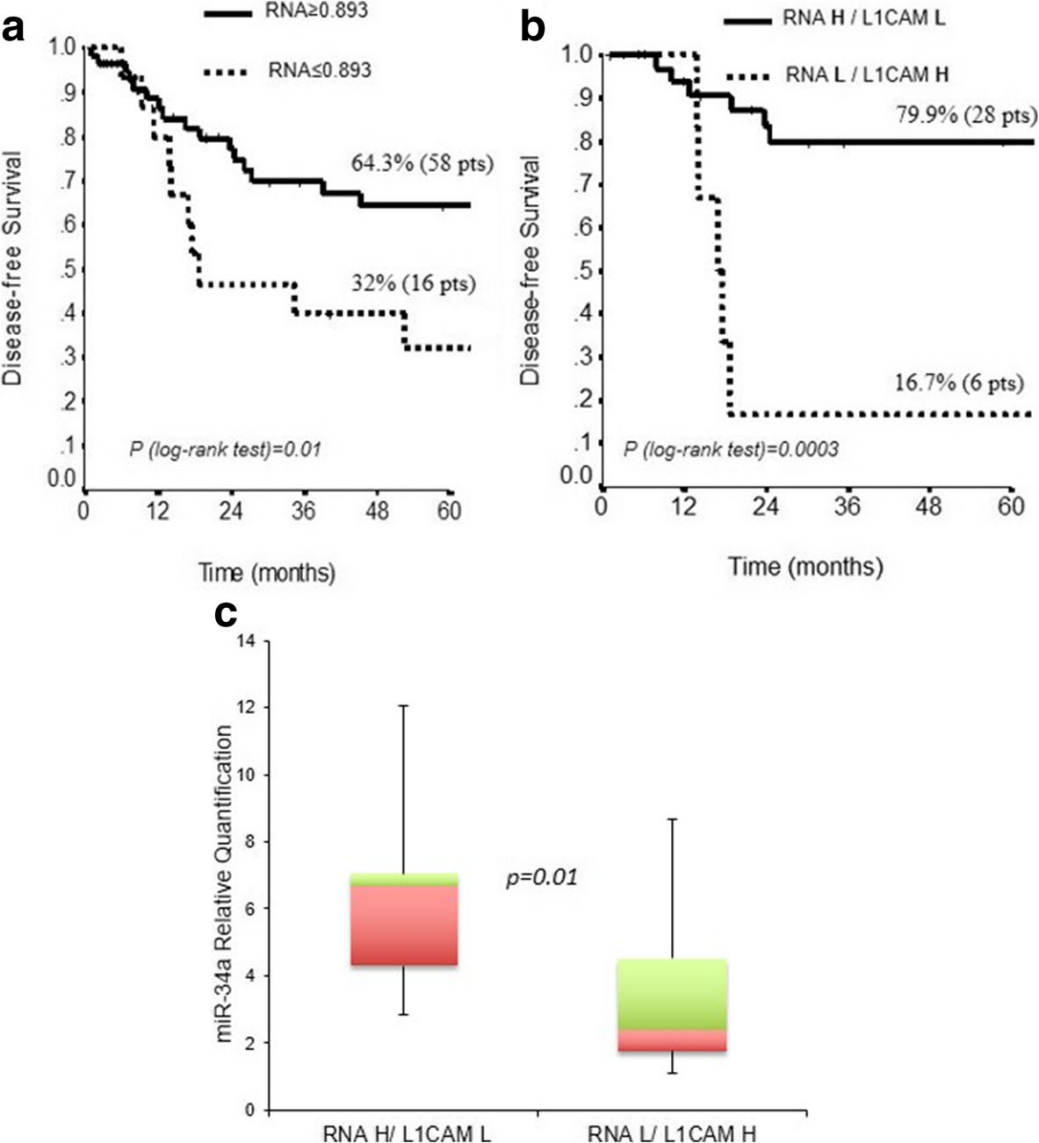
DFS according to L1CAM mRNA and protein expression in EC specimens. The box plot represents the distribution of miR-34a in the two subgroups of patients. **a** Kaplan-Meier estimate DFS for the expression of low vs high L1CAM mRNA level (P = 0.01). **b** Kaplan-Meier estimate DFS for the expression of mRNA H/L1CAM L vs mRNA L/L1CAM H (*P* = 0.0003). **c** In the Box Plot are reported the miR-34a values in the two subgroups of patients (mRNA L/L1CAM H and mRNA H/L1CAM L) (P = 0.01)

When we applied an explorative analysis, by RT-qPCR, we found that low level of L1CAM mRNA was expressed in recurrent compared to non-recurrent EC (*P* = 0.01) (Fig. 5). Based on the ROC curve, optimal cut-off for mRNA was determined at 0.893 (Additional file 2: Figure S2). The 5-years DFS was 64.3% for patients with high mRNA and 32% for lower mRNA (Fig. 6a). Thus, we correlated the expression of L1CAM protein with the corresponding mRNA level and identified two subgroups of patients with low mRNA/highL1CAM (mRNA L/L1CAM H) or high mRNA/lowL1CAM (mRNA H/L1CAM L) with different prognosis (Fig. 6b). The correlation of these data with DFS identified a subgroup of patients with mRNA L/L1CAM H protein that showed lower DFS (*P* = 0.0003) suggesting a post-transcriptional regulation of L1CAM protein.

Thus, we analyzed the level of miR-34a in these subgroups of patients and found that miR-34a was down regulated in patients with mRNA L/L1CAM H and accumulated in patients with mRNA H/L1CAM L confirming that miR-34a controls L1CAM protein expression (*p* = 0.01, Fig. 6c). Surprisingly, we did not identify a subgroup of patients with mRNA L/L1CAM L (data not shown).

## Discussion

Our study showed that L1CAM is highly expressed in vivo in recurrent compared to non-recurrent EC. We identified two groups of patients with different prognosis: high L1CAM expression and G3 with poor prognosis; low L1CAM expression and age lower than 67 years with good prognosis. Furthermore, we also found a subgroup of patients with low miR-34a/high L1CAM that showed lower DFS (*P* = 0.0003).

Although EC is characterized by a good prognosis, several authors, especially for early-stage EC, have reported a great heterogeneity of disease response [9]. EC site-specific recurrence patterns are influenced by classic prognostic factors such as histological type and grade, depth of myometrial invasion, LVSI, and nodal status [10, 11]. It is now well established that recurrences after primary surgical treatment are mostly located in the true pelvis with events generally occurring in the regional pelvic lymph nodes or in the vaginal vault [12]. However, other locations including distant metastases or peritoneal carcinomatosis can also be observed underlining the prognostic heterogeneity of the disease [13]. In fact, several randomized trials [14–16] have shown no improvement of DFS both with adjuvant radiotherapy and with lymphadenectomy. Despite debatable selection criteria and methodological bias, it is likely that negative results observed in these studies are mainly related to the natural history of the disease that is characterized by a pattern of relapse more similar to ovarian cancer than to cervical cancer.

In the era of personalized approach and innovative biotechnology, expression of L1CAM has been associated with aggressive subtypes of endometrial carcinoma [5]. Moreover, L1CAM has shown to be of great importance for the prediction of clinical outcome in FIGO-stage I, histologically confirmed endometrioid endometrial cancer (EEC) [17]. L1CAM has been extensively investigated in the last 15 years in relation to its capacity in enhancing cell motility and thereby promoting invasiveness and its expression has been reported be associated with many cancers including breast, gastric and colorectal cancers [18, 19].

The IHC detection of L1CAM in endometrial tumour samples is able to discriminate a subset of highly aggressive tumours with adverse clinical outcome and high risk of distant recurrences, and to assess the risk of pelvic lymph-node involvement.

To assess whether the expression of L1CAM in vivo was higher in recurrent compared to not-recurrent EC, we correlated the expression of the protein with the DFS. Our results confirm in a large single Institution series the prognostic role of L1CAM in EC patients. In our study, L1CAM seems to be highly predictive of tumour relapse in two groups of patients with opposite prognostic characteristics according traditional clinic-pathologic features:

- Patients < 67 years old, usually considered at better prognosis compared with older patients
- Poorly differentiated tumours (G3) typically considered at bad prognosis**.**

Advanced age, in EC, has been associated with a number of poor pathologic features including aggressive tumour histology, increased tumour grade, and deep myometrial invasion [20]. In addition, elderly women are more likely to be diagnosed with advanced stage disease [21, 22]. In our study EC low expressing L1CAM discriminate a group of patient with good prognosis when age lower than 67 years. High L1CAMs discriminate for a worse prognosis independently from age of patients.

High tumour grade and the presence of lymphovascular invasion remained as independent predictors of survival endpoints [23]. The association between L1CAM expression and higher grade has been shown by two recent reports including endometrial cancer of various histology and stages [24, 25]. Our study confirmed that L1CAM expression of the tumour is associated with poor differentiation and patients with L1CAM positive tumours were more likely to belong to groups of higher risk of relapse. Some authors suggest that grade 3 EEC may be more suitably considered non endometrioid endometrial cancer (NEEC) because they demonstrate similar immunohistochemical features and survival profile as those endometrial cancers traditionally considered more aggressive [26]. However, because the molecular profile of grade 3 EEC has not yet been fully characterized, it does not clearly correspond to either definition of type I and II cancer [27]. A recent multicenter study of L1CAM-expression in 1021 histologically confirmed EEC demonstrated L1CAM positivity in 17.7% and demonstrated that L1CAM-expression in EEC was an independent predictor of clinical outcome. A small percentage of these cases showed areas of non endometrioid differentiation in less than 10% of the tumor, and this was associated with L1CAM-expression [28]. This and others studies suggested that L1CAM expression carries prognostic value for histologically classified EEC and supports the identification of tumors with a NEEC component [29].

L1CAM promotes cell motility, invasion, chemo-resistance and metastasis formation. Elucidating genetic processes involved in the expression of L1CAM in cancers is of considerable importance. There is also increasing evidence that micro-RNAs can also have strong effects on gene expression [30]. Remarkably, it was previously reported in primary tumor sections an inverse correlation between L1CAM protein and miR-34a expression [8]. Our results demonstrate that L1CAM per se is a prognostic factor of EC progression. However, the analysis of L1CAM mRNA and protein expression identified two subgroups of patients with poor (mRNA L/L1CAM H) and good (mRNA H/L1CAM L) prognosis. Furthermore, the analysis of miR-34a in these two subgroups of patients, revealed that miR-34a was expressed at low level in the mRNA L/L1CAM H subgroup corresponding to patients with lower DFS, suggesting a rapid translation of the mRNA that results in a strong increase of the protein expression. Furthermore, we cannot exclude that the low mRNA found in these tumors could be due to a rapid mRNA degradation. As expected, the miR3-4a was accumulated in mRNA H/ L1CAM L subgroup of EC patients with good prognosis; in these tumors the accumulation of the mRNA could be due to the high level of miR-34a that inhibits its translation maintaining high level of mRNA and low level of L1CAM protein. Our findings demonstrate that L1CAM and the miR-34a expression have a marked prognostic significance identifying specific subgroup of patients that might be directed with different therapeutic protocols.

## Conclusion

The risk estimation in endometrial cancer is based on both preoperative and postoperative factors. In pre-operative setting L1CAM could be a useful additional tool. It could help to identify those EEC-patients who are at high risk of disease progression. Prospective studies may be needed to elucidate the value of L1CAM in predicting distant metastasis and to further investigate the effect of chemotherapy in patients with L1CAM positive tumors.

## Additional files

Additional file 1: **Figure S1.** ROC curve identify the optimal cut off point for the expression of L1CAM (20%). (TIF 99 kb)

Additional file 2: **Figure S2.** ROC curve identify the optimal cut off point for the expression of L1CAM mRNA (0.893). (TIF 99 kb)

## Abbreviations

DSF: Disease free survival
EC: Endometrial Cancer
EEC: Endometrioid endometrial cancer
FFPE: Formalin-fixed paraffin embedded
IHC: Immunohistochemical
L1CAM: L1-cell adhesion molecule
LVSI: Lymph vascular space invasion
NEEC: Non endometrioid endometrial cancer
