# Supplementary figures and images for: Endometrial cancer prognosis correlates with the expression of L1CAM and miR34a biomarkers
Source: J Exp Clin Cancer Res. 2018 Jul 6;37:139. doi: 10.1186/s13046-018-0816-1 (PMC6035393; doi:10.1186/s13046-018-0816-1)

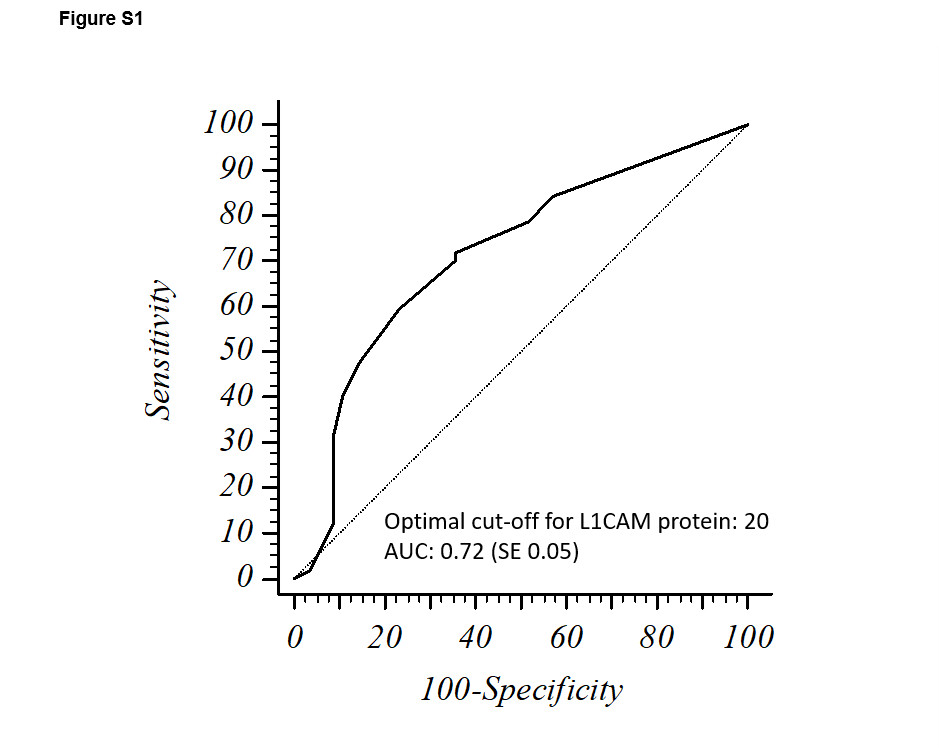

Supplement: Supplementary file 1 — Figure S1. ROC curve identify the optimal cut off point for the expression of L1CAM (20%). (TIF 99 kb) [file 13046_2018_816_MOESM1_ESM.tif]

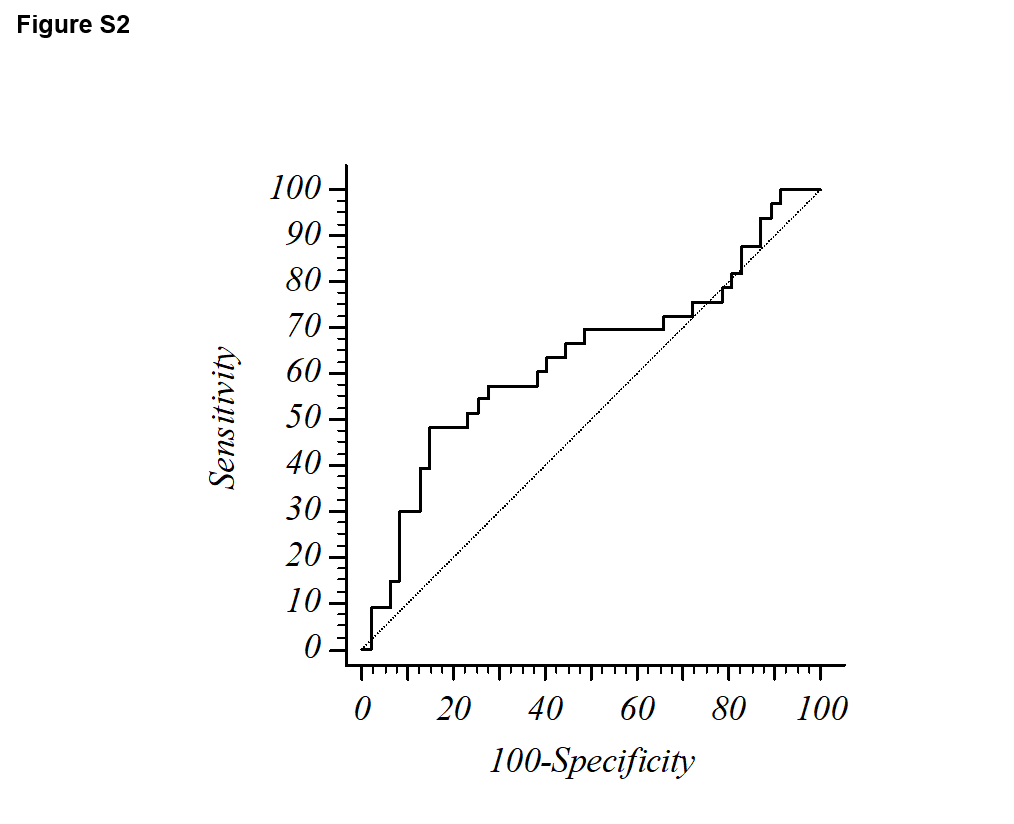

Supplement: Supplementary file 2 — Figure S2. ROC curve identify the optimal cut off point for the expression of L1CAM mRNA (0.893). (TIF 99 kb) [file 13046_2018_816_MOESM2_ESM.tif]
